# Eosinophilic Granulomatosis With Polyangiitis Gradually Worsening With Consecutive Pregnancies: A Case Report

**DOI:** 10.7759/cureus.54832

**Published:** 2024-02-24

**Authors:** Ryuichi Ohta, Taichi Fujimori, Chiaki Sano

**Affiliations:** 1 Communiy Care, Unnan City Hospital, Unnan, JPN; 2 Internal Medicine, Shimane University, Izumo, JPN; 3 Community Medicine Management, Shimane University Faculty of Medicine, Izumo, JPN

**Keywords:** general physician, rural, eosinophilia, mononeuritis multiplex, immunoglobulin e, pregnancy complications, vasculitis, eosinophilic granulomatosis with polyangiitis

## Abstract

This case report elucidates the diagnosis and management of eosinophilic granulomatosis with polyangiitis (EGPA), a form of systemic vasculitis, in a 32-year-old female presenting with progressive dermal, respiratory, and gastrointestinal symptoms following multiple pregnancies. The patient's history of allergic reactions and pregnancies suggested a gradual progression of EGPA, a condition rarely associated with pregnancy. Initial symptoms were misinterpreted as allergic reactions and acute gastroenteritis, delaying the correct diagnosis. Laboratory findings included eosinophilia and elevated immunoglobulin E, while further investigations ruled out other differential diagnoses, such as chronic eosinophilic leukemia. A clinical diagnosis of EGPA was made based on symptom progression, eosinophilia, and mononeuritis multiplex, absent typical granulomatous changes in the skin biopsy. Treatment with high-dose prednisolone and rituximab halted disease progression and improved symptoms, highlighting the critical importance of prompt diagnosis and treatment in preventing irreversible complications. This case emphasizes the need for general physicians to consider pregnancy as a potential trigger for autoimmune diseases like EGPA, especially in patients presenting with multi-symptom allergic reactions and high inflammatory markers.

## Introduction

Eosinophilic granulomatosis with polyangiitis (EGPA) is a rapidly progressive disorder based on the pathophysiology of systemic vasculitis [1]. This disorder can develop critical complications in the heart, lungs, and nerves [1]. Prompt diagnosis and treatment of vasculitis can prevent the progression of critical complications [2]. However, some cases can progress gradually, so clinicians may overlook the initial development of EGPA, just showing allergic reactions such as allergic dermatitis [3].

Autoimmune diseases can be triggered by pregnancy, but such cases of EGPA are not reported frequently. In addition, multiple pregnancies can progress the pathological condition of EGPA gradually [4]. This time, we have experienced a 32-year-old female with the chief complaints of progressive dermal, respiratory, and gastrointestinal symptoms following multiple pregnancies, diagnosed eventually with EGPA. The critical diagnosis and treatment were prompt, leading to the prevention of critical symptoms of mononeuritis multiplex. This case report shows the importance of pregnancy, followed by allergic symptoms, for the diagnosis of EGPA and prompt clinical diagnosis and treatment to prevent critical complications.

## Case presentation

A 32-year-old female came to a rural community hospital with chief complaints of nausea, vomiting, and diarrhea for one week. Seven days before the visit, she had intermittent epigastric pain during the day. Six days before the visit, the patient's nausea and watery diarrhea started, causing appetite loss. She went to a primary care doctor and was diagnosed with acute gastroenteritis, and probiotics were prescribed. Her abdominal pain and diarrhea were exacerbated. So, she came to our hospital. The past medical histories were allergic rhinitis, bronchial asthma, and allergic dermatitis. These diseases developed consecutively after her multiple pregnancies. She had four children. After her second pregnancy (she was 24 years old), she developed bronchial asthma. After her third pregnancy (she was 27 years old), she developed allergic rhinitis. After her fourth pregnancy (she was 31 years old), she developed allergic dermatitis. The medications were nasal mometasone, oral levocetirizine, oral montelukast, and fluticasone/umeclidinium/vilanterol inhaler.

The vital signs at the visit were as follows: blood pressure, 145/100 mmHg; pulse rate, 108 beats/min; body temperature, 37.5 °C; respiratory rate, 16 breaths/min; and oxygen saturation, 97% on room air. The patient was alert to the time, place, and person. Her physical examination showed an expiratory wheeze on chest sounds, increased intestinal peristalsis sounds, mild abdominal percussion tenderness without rebound tenderness, and eczema on extremities. No other abnormal neurological findings were noted. The laboratory test showed eosinophilia and systemic inflammatory conditions with increased immunoglobulin G and E (Table 1).

**Table 1:** Initial laboratory data of the patient eGFR: estimated glomerular filtration rate; CK: creatine kinase; CRP: C-reactive protein; Ig: immunoglobulin; HCV: hepatitis C virus; SARS-CoV-2: severe acute respiratory syndrome coronavirus 2; HIV: human immunodeficiency virus; HBs: hepatitis B surface antigen; HBc: hepatitis B core antigen; C3: complement component 3; C4: complement component 4; MPO-ANCA: myeloperoxidase antineutrophil cytoplasmic antibody; PR3-ANCA: protease 3 antineutrophil cytoplasmic antibody; CCP: cyclic citrullinated peptide; S/CO: sample/cut off

Parameter	Level	Reference
White blood cells	30.50	3.5–9.1 × 10^3^/μL
Neutrophils	33.4	44.0–72.0%
Lymphocytes	5.4	18.0–59.0%
Monocytes	3.2	0.0–12.0%
Eosinophils	57.8	0.0–10.0%
Basophils	0.2	0.0–3.0%
Red blood cells	4.55	3.76–5.50 × 10^6^/μL
Hemoglobin	12.1	11.3–15.2 g/dL
Hematocrit	37.8	33.4–44.9%
Mean corpuscular volume	84.0	79.0–100.0 fl
Platelets	38.2	13.0–36.9 × 10^4^/μL
Erythrocyte sedimentation rate	41	2–10 mm/hour
Total protein	8.5	6.5–8.3 g/dL
Albumin	3.1	3.8–5.3 g/dL
Total bilirubin	0.9	0.2–1.2 mg/dL
Aspartate aminotransferase	24	8–38 IU/L
Alanine aminotransferase	28	4–43 IU/L
Alkaline phosphatase	211	106–322 U/L
γ-Glutamyl transpeptidase	32	<48 IU/L
Lactate dehydrogenase	219	121–245 U/L
Blood urea nitrogen	10.3	8–20 mg/dL
Creatinine	0.52	0.40–1.10 mg/dL
eGFR	90.0	>60.0 mL/min/1.73m^2^
Serum Na	137	135–150 mEq/L
Serum K	4.4	3.5–5.3 mEq/L
Serum Cl	99	98–110 mEq/L
CK	35	56–244 U/L
CRP	13.13	<0.30 mg/dL
TSH	1.10	0.35–4.94 μIU/mL
Free T4	1.2	0.70–1.48 ng/dL
IgG	2403	870–1700 mg/dL
IgM	185	35–220 mg/dL
IgA	237	110–410 mg/dL
IgE	1752	<173 mg/dL
HBs antibody	0.67	mIU/mL
HBc antibody	0.00	S/CO
HCV antibody	0.00	S/CO
SARS-CoV-2 antigen	-	
anti-nuclear antibody	40	<40
C3	180	86–164 mg/dl
C4	43	17–45 mg/dl
MPO-ANCA	<1.0	<3.5 U/ml
PR3-ANCA	<1.0	<3.5 U/ml
anti-CCP antibody	<0.6	<5 U/ml
Urine test		
Leukocyte	Negative	Negative
Nitrite	Negative	Negative
Protein	Negative	Negative
Glucose	Negative	Negative
Urobilinogen	normal	
Bilirubin	Negative	Negative
Ketone	Negative	Negative
Blood	Negative	Negative
pH	6.0	
Specific gravity	1.039	

**Figure 1:**
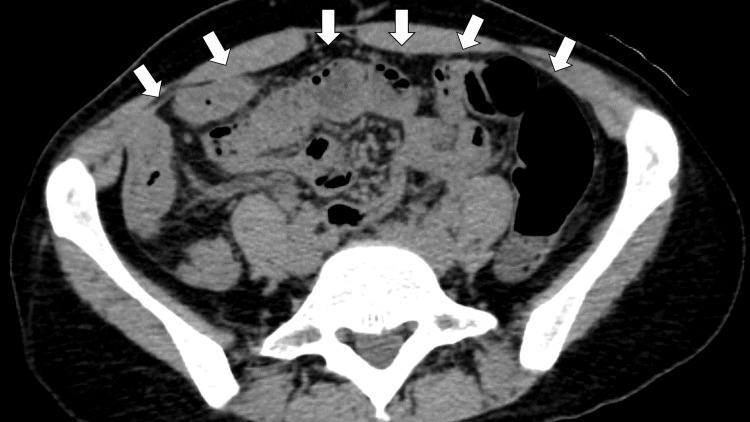
Abdominal computed tomography clarifying whole edematous changes in the small intestine (white arrows)

To investigate peritonitis, abdominal computed tomography clarified whole edematous changes in the small intestine (Figure 1).

Initially, she was diagnosed with asthma exacerbation with hypereosinophilic syndrome and treated with prednisolone at 25 mg/day daily. 

**Figure 2:**
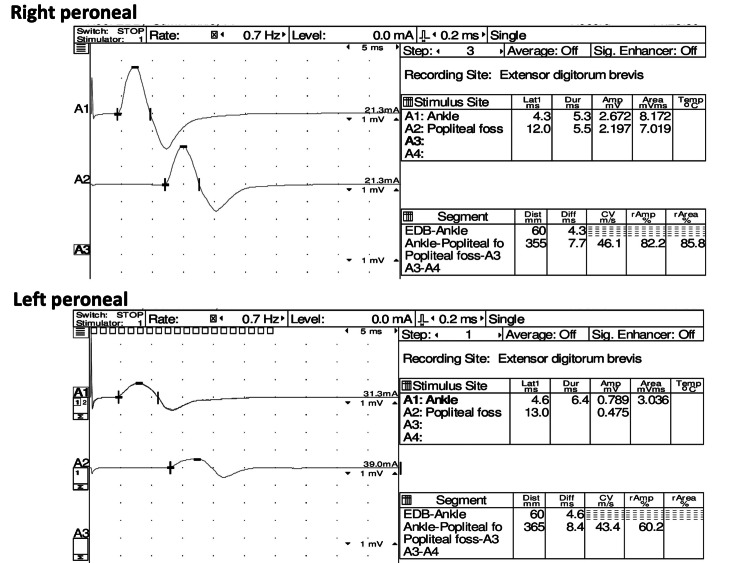
The bilateral peroneal nerve conduction velocity tests revealed possible demyelination and axonal injury, mainly in the left peroneal nerve

Two weeks after the initial treatment, her nausea, vomiting, and diarrhea improved without wheezing in the chest. However, her physical examination shows weakness in her left leg muscles, such as the tibialis anterior muscle and the extensor hallucis longus. The following laboratory test showed persistent eosinophilia and elevated C-reactive protein. The bilateral peroneal nerve conduction velocity tests revealed possible demyelination and axonal injury, mainly in the left peroneal nerve (Figure 2).

Suspecting chronic eosinophilic leukemia, peripheral blood sampling and DNA tests for platelet-derived growth factor receptor β (PDGFRB) 5q32 and fibroblast growth factor receptor 1 (FGFR1) 8p11.2 translocation were performed, clarifying no abnormalities. The skin biopsy of allergic dermatitis showed no granulomatous changes typical for EGPA, just the invasion of eosinophils in the skin.

Based on the clinical presentation of gradual exacerbating symptoms related to eosinophilia and mononeuritis multiplex with past medical histories of allergic rhinitis and asthma, she was clinically diagnosed with EGPA. The dose of prednisolone increased to 50 mg/day, and rituximab at 500 mg/week for four weeks was started. After the treatment, the progression of her leg weakness stopped, and it improved in two weeks compared to the previous condition. Eosinophil (234/μL) and immunoglobulin E levels (106 mg/dL) were normalized after four weeks of treatment with rituximab. The dose of prednisolone was tapered biweekly to 5 mg after four months.

## Discussion

This case report shows the gradual progression of EGPA following each pregnancy episode. As the pregnant state can trigger multiple immunological reactions in human bodies, general physicians should consider the pregnancy episode as a trigger for autoimmune diseases such as EGPA in young women with multiple symptoms related to allergic disease and high inflammatory conditions. The prompt investigation and treatment for EGPA should be considered through the differential diagnosis of critical diseases.

An adequate diagnosis of EGPA can be supported by taking a history of pregnancy and the related symptoms regarding rash and allergic reactions triggered by immunological reactions [5]. In the present case, the patient’s symptoms become progressive after each pregnancy. The pregnant state can trigger multiple immunological reactions in human bodies [6]. In pregnancy, immunological tolerance develops, accepting the presence of a fetus without immunological rejection [7]. However, immunological tolerance can preserve immune cells, which can react to themselves and eventually develop autoimmunity. The previous report shows that pregnancy can trigger the exacerbation of rheumatoid arthritis and systemic lupus erythematosus [8,9]. On the other hand, EGPA may not be as common as an autoimmune disease triggered by pregnancy [4]. Pregnancy can also trigger immunoglobulin E-mediated reactions [10]. Thus, EGPA, a pathophysiology that involves consecutive allergic reactions to oneself, can be triggered by consecutive pregnancies. However, there is a lack of evidence that terminating pregnancy can mitigate the exacerbation of EGPA. As general physicians usually care for pregnant patients, they should consider the pregnancy episode as a trigger for autoimmune diseases such as EGPA in young women with multiple symptoms related to allergic disease and high inflammatory conditions [11].

Eosinophilic granulomatosis with polyangiitis (EGPA) can be progressive, and its critical damage needs time to recover or is irreversible, demanding diagnostic investigation and treatments. In our case, ruling out critical differential diagnoses, we started a high dose of prednisolone and rituximab for the remission of EGPA. It can cause various critical organ features, such as interstitial pneumonia, carditis, and mononeuritis multiplex [12]. Such critical complications, once developed, can increase patients’ morbidity and mortality, impinging on their quality of life in rural contexts [13-15]. This time, the skin biopsy showed no granulomatous changes typical for EGPA, just the invasion of eosinophils in the skin. However, clinical reasoning increased the possibility of EGPA and exceeded the treatment decision threshold for EGPA. The treatment of vasculitis can be performed without typical pathological findings to avoid irreversible conditions after ruling out other critical diseases.

Vasculitis shows various symptoms at early or late appearances, so general physicians should enhance their clinical reasoning flexibly and promptly in rural contexts. As this article shows, patients with vasculitis need clinical diagnosis because of the acute progression of critical symptoms and the loss of patients’ quality of life. In diagnosis and treatment, rural general physicians need to diagnose and decide on the treatment plan and effective follow-up methods, considering the rare presentations of other rare diseases [16,17]. In this case, prompt investigation and treatment for EGPA should be considered through the differential diagnosis of critical diseases in general medicine.

## Conclusions

This case report shows the importance of the clinical history of pregnancy and the triggered symptoms to diagnose EGPA in young female patients. Prompt diagnostic measures and treatments are required to prevent the progression of critical complications of EGPA. General physicians should be cautious about eosinophilia with high IgE after multiple deliveries suspecting EGPA.
